# Improving Depression, and Quality of Life in Patients with Type 2 Diabetes: Using Group Cognitive Behavior Therapy 

**Published:** 2017-10

**Authors:** Zahra Noroozi, Sajedeh Hamidian, Niloofar Khajeddin, Mahnaz Mehrabi Zadeh Honarmand, Yadollah Zargar, Homeira Rashidi, Behrooz Dolatshahi

**Affiliations:** 1 Department of Psychology, Shahid Chamran University, Ahvaz, Iran.; 2 Department of Clinical Psychology, University of Social Welfare and Rehabilitation Sciences, Tehran, Iran.; 3 Department of Psychiatry, Jundishapur University of Medical Sciences, Ahvaz, Iran.; 4 Health Research Institute, Diabetes Research Center, Ahvaz Jundishapur University of Medical Sciences, Ahvaz, Iran.

**Keywords:** *Type II Diabetes*, *Depression*, *Cognitive Behavior Therapy*, *Group Psychotherapy*

## Abstract

**Objective:** Depression is a chronic condition comorbid with diabetes type 2 that often remains untreated. Dealing with diabetes is a challenging task for patients and can lead to depression in long term. These two conditions have a negative influence on each other and on the individual’s quality of life. The purpose of this study was to investigate the effect of group cognitive behavior therapy on depression, quality of life in women with diabetes type 2.

**Method** **:** We conducted a clinical trial among 30 women with diabetes type 2 comorbid with depression. The women were divided randomly into the two groups of *intervention* and control. Each group consisted of 15 individuals. The intervention group received 10 sessions of group cognitive behavior therapy while the control group didn’t.

**Results:** The results suggested that group cognitive-behavior therapy decreased depression symptoms (F=72.17, p<0.001), and improved quality of life of the patients (F=8.82, p<0.05) of the intervention group compared to the control group.

**Conclusion: **The results shows that group cognitive behavior therapy can affect depression symptoms, and consequently patients’ quality of life with diabetes type 2.

Diabetes and depression are 2 chronic conditions of public health, which are increasingly growing and have a large burden on the patients’ lives and society (1, 2). According to Centers for Disease Control and Prevention (2011), Type 2 diabetes mellitus (T2DM) constitutes 90% to 95% of newly diagnosed cases of diabetes in adults (4). Type2 diabetes starts smoothly usually in overweight individuals and in adults older than 30 years, and its most common primary symptom is lack of symptom. so, diagnosis and treatment of this disease requires careful considerations(1). 

Living with diabetes is associated with emotional distresses such as difficulty related to the treatment regimen and self-care behaviors (1). It negatively affects patients’ quality of life, increases functional disability, and reduces life expectancy. Some studies reported that people with diabetes (either Type 1 or Type 2) are twice more vulnerable to depression than individuals without diabetes (   1 ,7). 

Several studies have suggested a range of 24% to 30% for depression in T2DM (2).

There are conflicting approaches about the relationship between diabetes and depression. Some researchers believe that depression results from diabetes, and some others state that depression is an important risk factor for progression of diabetes, through disturbances in health care behaviors that have widely been observed in depression. Moreover, biochemical changes related to depression such as hypercortisolemia, inflammation, and sympathetic nervous system activation mechanisms that impair insulin sensitivity and glucose metabolism may contribute to development of diabetes. (9).

Prognosis of both depression and diabetes in a comorbid condition is worsened compared to each of these diseases separately (10). Several studies revealed that psychological factors have a powerful influence on controlling diabetes (1, 13-15). Crews et al. (2016) discussed anout the potentially significant correlations between psychological factors, neuropathy, body mass index, and physical inactivity in patients with diabetes (3)

These psychological factors can interfere with the usual medical treatment, 14, 16). Thus, psychological interventions along with medical therapy are found to be much more influential and more stable than just medical treatment (17).

One proposed psychological method for diabetes treatment with noteworthy impact on psychological and behavioral factors (sleep hygiene, diet, and physical activity) involved in glucose regulation is cognitive behavior therapy (17-20).

Therefore, considering the adverse effects of diabetes mellitus on quality of life, patients need to learn the best method to care for themselves. Hence, the present study was conducted to find whether group cognitive behavior therapy could provide those women with Type 2 diabetes with self-care methods and increase their quality of life to overcome depression.

## Materials and Methods

This was a clinical trial study with a control group and pretest- posttest design. Convenience sampling method was used to select female patients with Type 2 diabetes who referred to Diabetes Association of Ahvaz, Iran. We selected 70 individuals aged 18 to 49 years, who had at least high school diploma education level. After explaining the study process to the participants, they were enrolled in the study and were invited to fill out the 13-item Beck Depression Questionnaire; those with scores higher than 5 were evaluated through a semi-structured interview by the psychiatrist of the project to detect the criteria for major depression disorder based on DSM-IV-TR. In addition, participants were excluded if they had axis II personality disorders, psychosis, or addiction that could prevent regular participation and attendance in the sessions. Finally, 35 participants were diagnosed with depression, among whom 1 participant, due to travel, 3 due to lack of coordination of the meeting sessions with their working time, and 1 due to unwillingness to cooperate, were excluded from the study. The remaining 30 participants were referred to Behavioral Sciences Research Center of Ahvaz to take the blood sugar test, and then they were asked to fill out the questionnaire of quality of life. Then, they were assigned into the case and control groups and the intervention was conducted on the case group, while the control group remained on the waiting list. 

*Measures*: 

1. 13-item Beck Depression Inventory: It is a shortened version of 21-item form (Beck, 1961), which was presented in 1972. The questions include concepts such as sadness, pessimism, frustration, dissatisfaction, guilt, self-hatred, self-destruction, seclusion, indecisiveness, self-image, problem in working, exhaustion, and appetite. It was validated among a sample of students of Ahvaz. Using Cronbach’s alpha method, the final coefficient of 0.89 and using bisection method, the final coefficient of 0.87 was reported. To ensure reliability, Sharifnia (2000) used the Beck Disappointment Questionnaire and reached the reliability coefficient of 0.92(21).

2. World Health Organization Quality of Life (WHOQOL): This questionnaire includes 26 items in Likert scale ranging from 1 to 5. The questions evaluate emotions and behaviors of patients in the 2 recent weeks in aspects including hygiene and physical health, psychology, social relations, and social environment (22). For each area, scores from 4 to 20 can be achieved, representing the worst and the best condition in each area, respectively. Theses scores can be converted into scores ranging from 0 to 100. To ensure the reliability of the scale, Nasiri (2006) used 3 methods: retest (with 3 weeks interval), bisection, and Cronbach's alpha, the reliability of each was 0.67, 0.87, and 0.84, respectively (23).

*The Intervention Program*

The guidelines of the intervention program were arranged based on “Cognitive Behavioral Group Therapy for Adolescents with Type I Diabetes.” by Wildermuth (2008) and consisted of 10 sessions each lasting for 1.5 hours. A summary of the sessions’ content is demonstrated in Table 1(3).

*Statistical Analysis* *:*

Before conducting ANCOVA, to ensure that the variables had the assumptions underlying a covariance analysis, Klomograf-Smirnov test was conducted, which showed normal distribution of the variables (Z>0.05). Levin test results revealed no significant difference between group variance in homogeneity of the variance (P>0.05). 

## Results

Data analysis was performed on data gathered from 30 participants (15 individuals in each group) using SPSS software Version 16. Prescriptive data are depicted in Table 2. The mean score of depression severity and quality of life in posttest significantly improved compared to pretest in the case group after the intervention, a trend that was not seen in the control group. The results of ANCOVA in Table 3 demonstrate that the intervention provided to the case group resulted in a significant difference between the 2 groups. According to Table 2, the case group's mean scores of depression and quality of life have considerably decreased compared to those of the control group; and according to Table 3, the difference was statistically significant at p = 0.001 and p = 0.006, respectively. The statistical power of both tests was equal to 1, meaning that the results can be attributed to the effect of the intervention not errors.

The effect size in Table 3 indicates that 0.73 and 0.25 of the difference between the 2 groups in depression and quality of life, respectively, was due to the intervention.

**Table 1:** Content of the group cognitive behavior therapy Sessions

**Session**	**Content**
**Session 1**	Introduction and definition of the therapy and disease, objectives, and rules of the group
**Session 2**	The relationship between thoughts, feelings and behavior, dysfunctional thinking styles, cognitive errors, and homework
**Session 3**	Cognitive restructuring, identifying symptoms and thoughts associated with depression, and homework
**Session 4**	Chain of antecedents, responses and consequences, breaking the destructive chain
**Session 5**	Information about carbohydrates metabolism, the differences between Type I and II diabetes, nutrition and exercise, and benefits of exercise for patients with diabetes
**Session 6**	Definition of assertiveness, negative thoughts and self- talking that prevents assertion; the difference between passivity, aggression, and assertiveness
**Session 7**	Managing impulsivity, self-control strategies, enhancing mood and increasing pleasant events
**Session 8**	Stress, stressors and management, problem- solving, relaxation training
**Session 9**	Self-esteem, the negative impact of self-evaluation on self-esteem
**Session 10**	Planning for relapse prevention, and the need to practice the acquired skills

**Table 2:** The Mean and Standard Deviation of the Study Variables in the Case and Control Groups Before and After the Group Cognitive Behavior Therapy

**Group**	**Case**		**Control**	
	**Pretest**	**Posttest**	**Pretest**	**Posttest**
**Statistics** **Variable **	Mean(SD)	Mean(SD)	Mean(SD)	Mean(SD)
**Depression**	11.4(4.08)	3.53(1.4)	9.13(3.88)	9.93(3.05)
**QOL**	74.6(7.56)	81.3(10.39)	73.73(6.67)	71(5.26)

**Table 3:** Covariance Analysis on the Mean Scores of the Posttest for Depression and Quality of Life Variables

**Variable**	**Source of** **change**	**Sum of ** **squares**	**Degree of ** **freedom**	**Mean ** **squares**	**F coefficient**	**Effect size**	**Statistical ** **power**	**P-** **value**
**Depression**	Pretest	30.48	1	30.48	6.7	0.20	0.70	0.016
	Group	328.24	1	328.24	72.17	0.73	1	0.001
	Error	118.24	26	4.54				
**QOL**	pretest	18.99	1	18.99	0.41	0.01	0.09	0.526
	Group	405.17	1	405.17	8.82	0.25	1	0.006
	Error	1193.9	26	45.92				

QOL = Quality of Life

## Discussion

The results of the present study about the effectiveness of group cognitive behavior therapy on depression and quality of life in women with Type 2 diabetes is in line with the study of Esbitt et al.(2015), Bastelaar (2008) and Skinner (2002). In their studies, they found that the effect of psychological interventions, specifically cognitive-behavioral ones, on patients with diabetes could decrease depressive symptoms and diabetes-specific distress on one the hand and could improve the patient’s adherence to medical treatment on the other hand (24-26).

As it was depicted in the results, the case group scores of depression and quality of life was significantly improved after the intervention; and this could be attributed to cognitive behavioral interventions, which has broadly endorsed to be effective on primary and secondary depressive symptoms (27, 28). Diabetes-specific emotional distress was targeted by training skills such as stress management, problem solving, and skills for interpersonal interactions. The case group participants were also given information about sugar metabolism and healthy nutrition for Type 2 diabetes. 

Increasing the quality of life of the case group patients after the intervention could be related to decreasing the emotional burden of diabetes, which increased the patients’ adjustment; and consequently, improved their quality of life (29). Approaches to the quality of life can be considered it in both objective and subjective aspects. The objective dimension includes environmental conditions and access to health services and information. Because this dimension remained unchanged in the present study, the most impacts were seen in the subjective and attitude areas. Among the subscales of the quality of life, social relationships and physical health have mostly benefited from the intervention, which could be due to applying anger management, assertiveness, and impulse control in interpersonal relationships. 

About the potential underlying mechanisms that link diabetes to depression, Moulton et al. (2015) enumerated psychological and physiological factors. Among psychological factors, dysfunctional assumptions about the disease such as “Will my diabetes condition get worse gradually whether I use medications or not” could have considerable direct and indirect impacts on diabetes (30). 

The assumption of cognitive behavioral theory is that the way people think affects their behavioral choices. For example, when blood sugar and nutritional and medical diets control in these individuals is disrupted, cognitive distortions of those who have little self-control lead to feeling of futility of medical efforts and hopelessness. Hence, they are more likely to drop out of treatment and show poor adherence. Following poor treatment adherence, metabolic dysregulation would occur that endorses the cognitive distortions and aggravates the existing dysfunctional thoughts (31). Cognitive-behavioral therapy facilitates the encountering of the patient with his conflicts and guides him to break such kinds of vicious circles.

It is a matter of debate whether depression and diabetes-specific emotional distress in diabetic patients are a unit component or different entities that must be dealt with separately. Fisher et al. have assumed an underlying construct for both of them (32). Some other theorists considered these 2 factors to be isolated from each other (33). It seems that regardless of which of this hypothesis is a better description of the nature of the relationship, treatment should focuse on stress management and self-care behaviors associated with the disease as well as cognitive and behavioral interventions related to depression. Thus, the intervention group in this study received information about carbohydrates and sugar metabolism in Type 2 diabetes.

As studies suggest that stress could mediate the link between cognitive distortions and adherence to treatment(31), interventions in diabetes should address several domains including depression and diabetes-specific emotional distress, which is related to glycemic control(1, 33).

Endevelt et al. stated that both individual and group therapy are equally influential in achieving outcomes in controlling diabetes and given that group therapy is more cost effective, it is preferable(34, 35). In the present study, using group cognitive behavior therapy and through normalizing and evaluating evidences, the participants observed the behavior of others with the same problem, and as a result, their dysfunctional thoughts about the disease and considering diabetes as weakness and inadequacy were changed.

The use of such psychological treatments for patients with health problems on the one hand increases their compliance with the medical therapy, and on the other hand, with improving the psychological aspects related to the issue, it facilitates the healing process. Psychological treatments also help improve the quality of life despite the disease through increasing patients’ acceptance about the symptoms.

## Limitation

Because of using convenience sampling, the results of this study should be generalized with caution. To control the nonspecific effects of the psychotherapy method, the design of the present study could be replicated by adding another group who receive a different method of psychotherapy. It is suggested that this study be replicated by using a large sample size, multi-center, cohort design, and subtype classifications of patients. Conducting the study only on females was another limitation of the present study.

## Conclusion

Cognitive-behavioral therapy in depression comorbid with T2DM, makes considerable changes in the lifestyle and to the ways dealing with the disease. Given the interaction between depression and diabetes, such interventions target the portion of the disease progress, which is related to psychological conditions such as depression. It is the breaker of the chain of such interactions that is often seen in chronic cases. Group performance of psychotherapy, in addition to saving time and money, provides an opportunity for people to share their experiences about coping with such difficult situations, and it facilitates the treatment process through the effects of observational learning and increased acceptance.
